# Circular RNA_0062582 promotes osteogenic differentiation of human bone marrow mesenchymal stem cells via regulation of microRNA-145/CBFB axis

**DOI:** 10.1080/21655979.2021.1921553

**Published:** 2021-05-23

**Authors:** Feng Li, Hao Wu, Guoyou Zou, Dingwei Cang, Yixin Shen

**Affiliations:** a Department of Orthopedics, The Second Affiliated Hospital of Soochow University, Suzhou, Jiangsu, P.R.China; b Department of Orthopedics, The First People’s Hospital of Yancheng, Yancheng, Jiangsu, P.R.China

**Keywords:** Circular rna, microRNA-145, osteoporosis, circ_0062582, differentiation

## Abstract

Osteoporosis poses a threat to human health worldwide. To date, there have been few studies regarding targeted treatment of osteoporosis. We aimed to identify the possible molecular mechanism of circular RNA (circ)_0062582 in osteogenic differentiation, and the interactions among circ_0062582, microRNA-145 (miR-145) and core-binding factor subunit β (CBFB). The proliferation of human bone marrow mesenchymal stem cells (hBMSCs) was tested with a cell counting kit-8 assay. Circ_0062582, miR-145 and CBFB were overexpressed by transient transfection. Dual-luciferase reporter assay system was used to analyze the combination among circ_0062582, miR-145 and CBFB. Additionally, the levels of circ_0062582, miR-145, CBFB, osterix (OSX), osteocalcin (OCN) and collagen type 1 (COL1) were detected by means of RT-qPCR or western blot analysis. Alkaline phosphatase and Alizarin red stainings were performed to analyze the degree of osteogenic differentiation under the control of circ_0062582, miR-145 and CBFB. The results demonstrated that circ_0062582 level was notably elvated during osteogenic differentiation of hBMSCs. Circ_0062582 overexpression significantly promoted osteogenic differentiation and upregulated the levels of osteogenic differentiation-related proteins, including OSX, OCN and COL1. In addition, miR-145, which was identified as the target gene of circ_0062582, could specifically target CBFB 3′-UTR regions. Next, these changes caused by the overexpression of circ_0062582 were reversed following the addition of miR-145 mimic. Following overexpression of CBFB, osteogenic differentiation was increased. In summary, these results demonstrated that the role of circ_0062582 in osteoporosis is mediated through regulating the expression level of CBFB via miR-145.

## GRAPHICAL ABSTRACT

**Figure:**
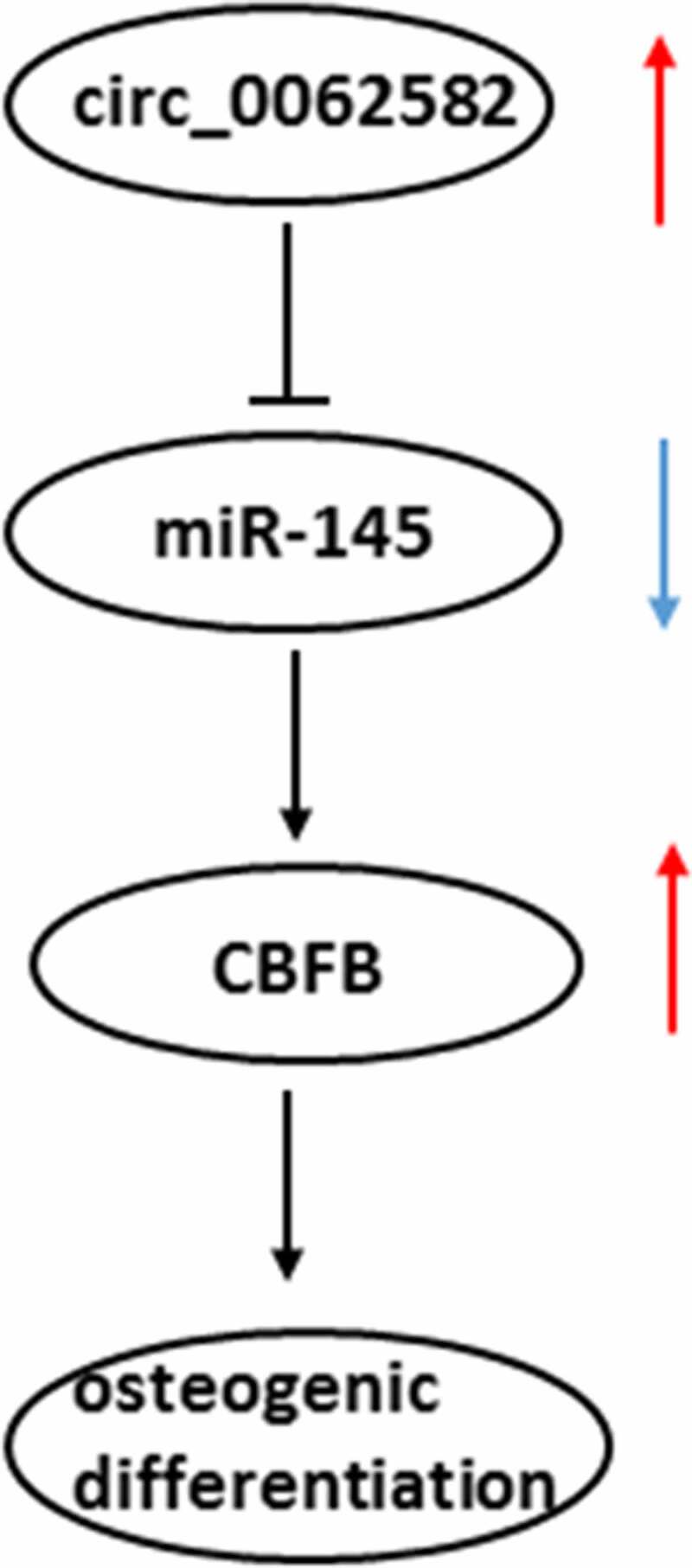

## Introduction

Osteoporosis is defined as a kind of bone disease characterized by reduced bone strength and enhanced risk of fracture. It is usually asymptomatic or accompanied by mild symptoms, which is not only a common cause of clinical pathological fracture, but also one of the high risk factors affecting human health [1]. Studies have shown that the worldwide incidence of osteoporosis is 15% in people over 50 years old and 70% in people over 80 years old [2,3]. At present, the number of patients with osteoporosis worldwide exceeds 200 million, while the number of patients is expected to increase to 300 million by 2023 as a result of aging of general population [4]. Currently, the therapeutic approaches for osteoporosis are limited and had the inevitable side effects [5,6]. Therefore, it is urgent to identify novel biomarkers for the detection and treatment of osteoporosis.

As a main hematopoietic organ of human body, bone marrow is composed of bone marrow hematopoietic stem cells, bone marrow adipose tissue and stromal cells [7]. Osteoblasts, which are differentiated from human bone marrow mesenchymal stem cells (hBMSCs), can exert significant roles in bone formation. The bone mass is continuously remodeled and maintained through a process involving a balance between bone formation and resorption. Bone loss occurs if this balance is disrupted and is mediated by osteoblasts (derived from mesenchymal stem cells) and osteoclasts (derived from hematopoietic precursors) [8,9]. Osteogenic differentiation is a key factor in bone regeneration. A growing body of literature has shown that dysfunction of bone remodeling and osteoblastic bone formation are involved in the pathological process of patients with osteoporosis [10]. Stimulating bone formation has been considered as an essential therapeutic approach for the treatment of osteoporosis. It is pointed out that compared with normal subjects, osteoblast differentiation in patients with osteoporosis is weaker than normal, which shows that the level of osteoblast differentiation is associated with the occurrence, development and evolution of the pathological process of osteoporosis [11]. Therefore, an in-depth study of the osteogenic differentiation process of hBMSCs will contribute to a better clarification of osteoporosis pathogenesis. Circular RNAs (circRNAs/circs), a novel class of non-coding RNAs with circular structure, are crucial molecular regulators implicated in multiple biological processes, including cell growth, migration and differentiation, that have great therapeutic potential [12–14]. A previous study has suggested that circRNAs may be used as biomarkers of osteoporosis, among which the level of circ_0062582 was reportedly to be markedly downregulated [15,16]. MiRNAs are non–coding endogenous RNAs that play inhibitory effects on translation by targeting mRNAs [17]. The possible link between circRNAs and miRNAs is proposed as evidenced by previous publications showing the changes in the course of various diseaes brought from the regulation of miRNAs via circRNAs [18]. Emerging evidence supports the notion that circ_0062582 expression is markedly downregulated in osteoporosis patients compared with the paired control samples [19]. The Encyclopedia of RNA Interactomes suggests that circ_0062582 can target microRNA (miRNA/miR)-145. One study has reported that estrogen may promote osteogenic differentiation via inhibiting miR-145 expression and regulating the level of osteoprotegerin after transcription [20]. In addition, Targetscan prediction shows that miR-145 could combine with core-binding factor subunit β (CBFB). CBFB is an essential binding chaperone to maintain the activity of osteoblast transcription factor Runx, which could regulate the bone development by stabilizing Runx family proteins [21]. However, no research has shown the role and potential mechanisms of circ_0062582, miR-145 and CBFB in osteoporosis.

The present study aimed to investigate the effects of circ_0062582 on osteogenic differentiation in cultured hBMSCs, and to clarify its regulatory effects on miR-145 and CBFB.

## Materials and methods

### Cell culture and treatment

Human bone marrow-derived BMSCs (hBMSCs) were the product of Inbiobank Stem Cell Bank (http://www.inbiobank.org). Cells were incubated in DMEM (ScienCell Research Laboratories, Inc.) containing 10% FBS (Beijing Solarbio Science & Technology Co., Ltd.) in a 5% CO_2_ incubator at 37°C. When hBMSCs were cultured to the fourth generation, cells were plated into a 6-well plate (1x10^6^ cells/well) that was pre-coated with gelatin. After 24 h, 2 ml osteogenic differentiation medium (OM) supplemented with 10% FBS, 1% glutamine, 10 nM dexamethasone, 0.2 nM ascorbic acid and 10 nM β-glycerophosphate was added to each well. Cells were collected on days 0, 7, 14 and 21. The present study was approved by the Ethics Committee of The First People’s Hospital of Yancheng (Yancheng, China).

## Cell transfection

For circ_0062582 overexpression, the full-length circ_0062582 cDNA product was amplified in hBMSCs and then cloned into the pLCDH-ciR (Shanghai GeneChem Co., Ltd.), which contained a front and back circular frame. Cells were transfected with the construct with Lipofectamine® 2000 (Invitrogen). Empty vector was used as a negative control. Subsequent experiments were performed 48 h following transfection. Moreover, miR-145 mimic and mimic negative control (miR-NC) were provided by Genepharma (Shanghai, China). Transfection experiments were carried out by means of Lipofectamine® 3000 (Invitrogen) following the manufacturer’s guide. For CBFB overexpression, pcDNA3.1-CBFB or pcDNA3.1-NC mock vector was transfected into hBMSCs with Lipofectamine® 2000 (Invitrogen) in accordance with the manufacturer’s recommendations. After transfection, CBFB level was determined with reverse transcription-quantitative PCR (RT-qPCR) analysis

## RT-qPCR analysis

Total RNA was obtained using TRIzol reagent (Invitrogen), and cDNA was synthesized with the PrimeScript RT Reagent Kit (Takara, Japan). Using cDNA as the template, the non-coding RNA and gene expression levels were analyzed by PCR conducted using iTaq™ Universal One-Step iTaq™ Universal SYBR® Green Supermix (Bio-Rad Laboratories, Inc.) on an ABI 7500 instrument (Applied Biosystems). All primers were provided by Genepharma (Shanghai, China). The results were calculated using the 2^−ΔΔCq^ method [22]. GAPDH and U6 served as the internal references.

## Cell viability assay

Cell Counting Kit-8 (CCK-8; Beyotime, China) assay was performed for determining the viability of hBMSCs. 5 × 10^3^ cells were seeded into the 96-well cell culture plates and maintained at 37°C. At each of the desired time points, plates were cultured for 4 h after each well was added with 10 μL CCK-8 reagent. The optical density was determined at 450 nm with a microplate reader (Bio-Rad Laboratories, Richmond, CA, USA).

## Alkaline phosphatase (ALP) measurement

ALP activity was measured by means of a commercially available ALP assay kit (Nanjing Jiancheng Bioengineering Institute). Briefly, solution No. 1 from the kit was added to the cells, which were incubated at room temperature for 2 min. Subsequently, cells were washed twice using PBS (Sigma-Aldrich, St. Louis, MO, USA) and added by ALP staining working solution for incubation at 37°C for 4 h. Images were captured under an Olympus BX-51 fluorescence microscope (Olympus Corporation).

## Alizarin red staining

The degree of calcium deposits was evaluated by Alizarin red staining. Briefly, cells were fixed using paraformaldehyde and then washed with PBS twice. Subsequently, cells were stained with 0.1% Alizarin red-TrisHCl (pH 8.3) solution for 30 min. Images were captured under a microscope.

## Luciferase activity reporter assay

The Circinteractome and StarBase online bioinformatics softwares were used to predict the targets of circ_0062582 and miR-145, and the results showed that miR-145 could be potentially targeted by circ_0062582 and that CBFB was identified as a putative target of miR-145. Dual luciferase activity reporter experiments were executed for verifying these combinations. Briefly, wild-type (WT) or mutant (MUT) circ_0062582 or CBFB binding miR-145 was subcloned into a pGL3 basic vector (Promega Corporation). Circ_0062582 WT/MUT or CBFB-3’ untranslated region (UTR) WT/MUT, miR-141-3p mimic or miR-NC were then co-transfected with cells with Lipofectamine 2000 (Invitrogen) following the manufacturer’s guide. Two days after transfection, the luciferase activities were analyzed with a Dual-Luciferase Reporter Assay Kit (Promega Corporation).

## Western blotting (WB)

Total proteins in cells were collected and lysed with RIPA Lysis Buffer (Beyotime). The concentration of protein was examined according to the BCA kit instructions (Kaiji, China). Afterward, 40 μg of each sample were loaded and separated by a 10% SDS-polyacrylamide gel. The proteins were bound to the PVDF membrane (Millipore) by electrophoretic means, and then the nonspecific sites were blocked on the membrane with 5% skimmed milk. Blots were probed with specific primary antibodies and secondary antibody conjugated with horseradish peroxidase. Protein bands were visualized with enhanced chemiluminescence substrate (Pierce, USA) using chemiluminescence imaging equipment (Claremont, CA, USA). The relative intensities of target bands were semi-quantified using ImageJ software and normalized by the intensity of GAPDH.

## Statistical analysis

Results were presented as the mean ± SD. Analysis of the data was carried out with GraphPad Prism 8.0 (GraphPad Software). For multiple comparisons, analysis of variance (ANOVA) with Tukey’s post hoc test was employed to analyze significance. P-values of <0.05 were taken as statistically significant.

## Results

### Overexpression of circ_0062582 promoted the osteogenic differentiation of hBMSCs

**Figure 1.**
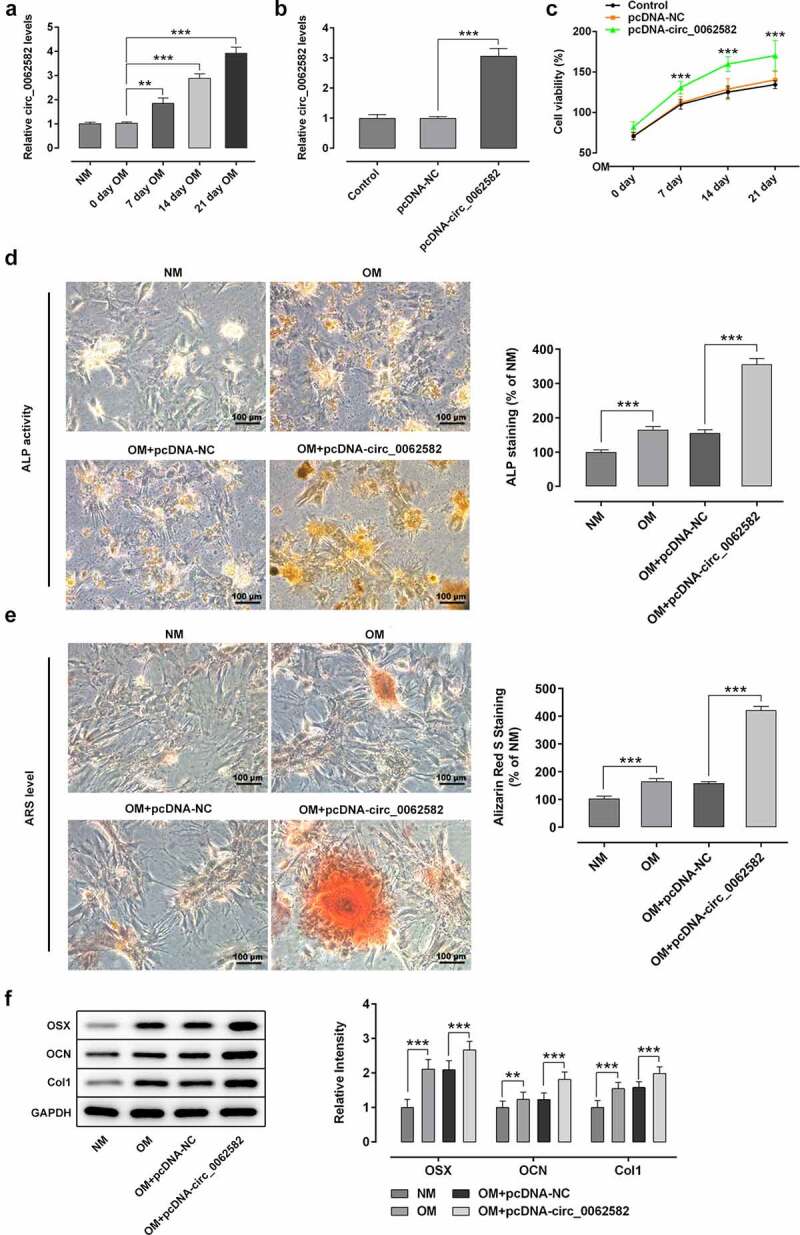
**Overexpression of circ_0062582 promoted the osteogenic differentiation of hBMSCs**. (a) Expression levels of circ_0062582 in hBMSCs was detected by RT-qPCR analysis. (b) Detection of the expression of circ_0062582 following addition of pcDNA-circ_0062582. (c) Viability of hBMSCs was measured using CCK-8 assay. (d) Alkaline phosphatase staining was used to detect the differentiation activity of hBMSCs. Magnification, x200. (e) Alizarin red staining was used to detect the mineralization levels of hBMSCs. Magnification, x200. (f) Protein expression levels of OSX, OCN and COL1 were tested by means of western blot assay. **P < 0.01 and ***P < 0.001. hBMSCs, human bone marrow mesenchymal stem cells; circ, circular RNA; OSX, osterix; OCN, osteocalcin; COL1, collagen type 1

It has been reported that circ_0062582 expression is significantly downregulated in osteoporosis patients compared with the paired control samples [19]. To determine the effect of circ_0062582 on osteoporosis, RT-qPCR was used to detect the level of circ_0062582 in hBMSCs. As exhibited in Figure 1a, circ_0062582 expression was conspicuously upregulated during osteogenic differentiation of hBMSCs. Subsequently, the circ_0062582 overexpression plasmid was constructed to explore the effect of circ_0062582 on osteoporosis. Compared with the pcDNA-NC group, the level of circ_0062582 was markedly enhanced in pcDNA-circ_0062582 group (Figure 1b). Next, CCK-8 assay was performed to analyze the effect of circ_0062582 overexpression on the proliferation of hBMSCs. As shown in Figure 1c, the cell differentiation levels in the pcDNA-circ_0062582 group were higher than those in the control group over time. In addition, Alkaline phosphatase and Alizarin red staining were performed to analyze the degree of osteogenic differentiation following the overexpression of circ_0062582. As displayed in Figure 1d, as comparison to the NM group, the presence of yellow precipitates indicated that the cells in the OM group were undergoing differentiation, and the markedly increased amount of yellow precipitates in the NC group after the addition of overexpression plasmid indicated that the overexpression of circ_0062582 could promote the differentiation of hBMSCs. This was also supported by the results of the Alizarin red staining experiments (Figure 1e). Results of western blotting revealed increased protein expression levels of OSX, OCN and COL1 in the overexpression group (figure 1f). Taken together, overexpression of circ_0062582 promoted the osteogenic differentiation of hBMSCs.

**Overexpression of miR-145 suppressed the promotion effect of circ_0062582 on the osteogenic differentiation of hBMSCs**

**Figure 2.**
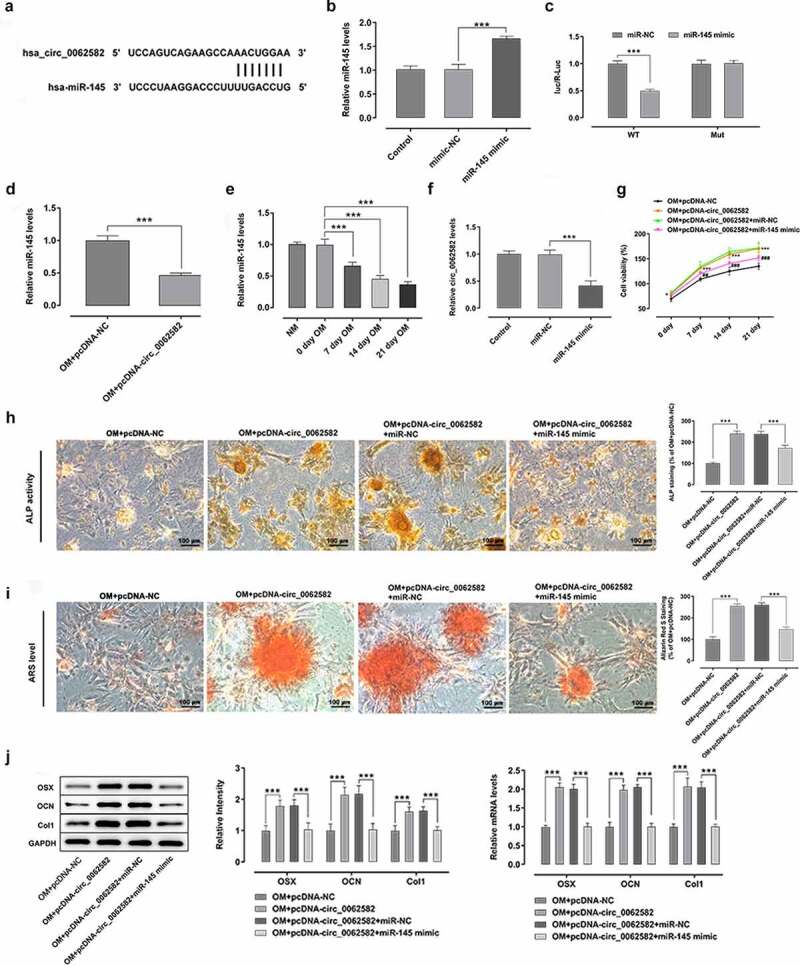
**Overexpression of miR-145 suppressed the promotion effect of circ_0062582 on the osteogenic differentiation of hBMSCs**. (a) Prediction of target genes. (b) MiR-145 expression was tested with RT-qPCR after transfection with miR-145 mimic. (c) Results of the Promega dual-luciferase reporter gene assay. (d) Expression of miR-145 in hBMSCs was examined using RT-qPCR. (e) The expression of miR-145 was evaluated with RT-qPCR after cells being induced by OM. (f) Detection of the expression levels of circ_0062582 following transfection with miR-145 mimic. ***P < 0.001. (g) Viability of hBMSCs. ***P < 0.001 vs. OM+pcDNA-NC and ^###^P < 0.001 vs. OM+pcDNA-circ_0062582+ miR-NC. (h) Alkaline phosphatase staining was used to detect the differentiation activity of hBMSCs. Magnification, x200. (i) Alizarin red staining was employed to evaluate the mineralization levels of hBMSCs. Magnification, x200. (j) Protein expression levels of OSX, OCN and COL1 were measured using western blot analysis. ***P < 0.001. miR-145, microRNA-145; hBMSCs, human bone marrow mesenchymal stem cells; OSX, osterix; OCN, osteocalcin; COL1, collagen type 1

CircRNAs can regulate multiple physiopathological processes by binding to miRNAs. MiR-145 was selected from the circinteractome prediction site as the targeted miRNA of circ_0062582 (Figure 2a). As displayed in Figure 2b, miR-145 expression was notably upregulated after transfection with miR-145 mimic relative to the miR-NC group. Then, the result of dual-luciferase experiments showed that circ_0062582 directly targeted miR-145 (Figure 2c). MiR-145 expression was assessed by RT-qPCR following overexpression of circ_0062582. It was found that the expression of miR-145 was decreased as time progressed (Figure 2d and 2e). Subsequently, RT-qPCR assay was employed to determine the effect of miR-145 overexpression on the expression of circ_0062582. As what is observable from figure 2f, miR-145 overexpression remarkably downregulated the expression circ_0062582 in miR-145 mimic group relative to the miR-NC group. Additionally, results of CCK-8 assay showed that the activity of hBMSCs in the NC group was decreased following transfection with miR-145 overexpression plasmid (Figure 2g). The results of ALP and alizarin red staining demonstrated that the cell differentiation levels were decreased following transfection with miR-145 overexpression plasmid (Figure 2h and 2i). The results of WB and RT-qPCR assays suggested that the levels of OSX, OCN and COL1 were downregulated in the OM + pcDNA-circ_0062582 + miR-145 mimic group as comparison to the OM + pcDNA-circ_0062582 + miR-NC group (Figure 2j). In summary, overexpression of miR-145 suppressed the promotion effect of circ_0062582 on the osteogenic differentiation of hBMSCs.

## CBFB was a direct target gene of miR-145 in hBMSCs

**Figure 3.**
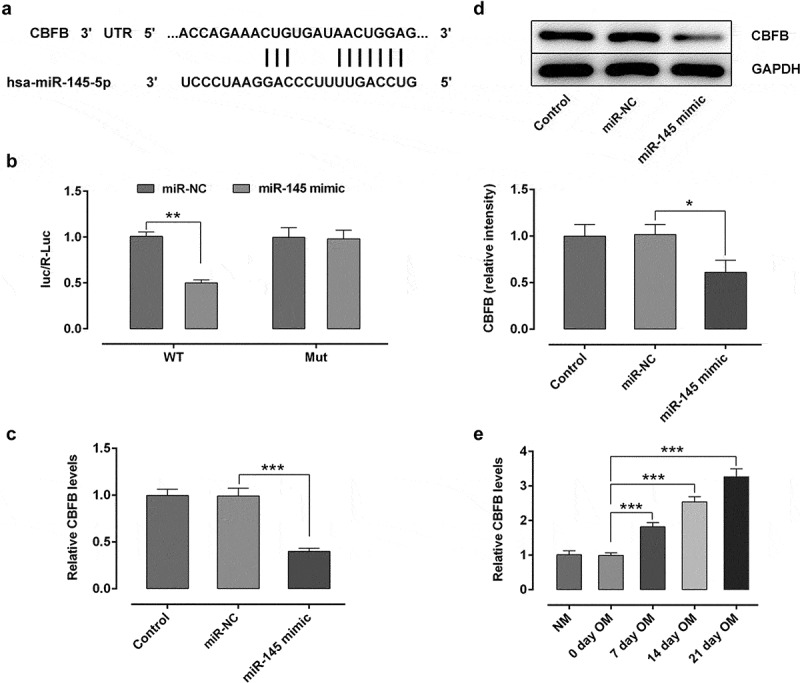
**CBFB was a direct target gene of miR-145 in hBMSCs**. (a) Prediction of target genes. (b) Results of the Promega dual-luciferase reporter gene assay. (c) Detection of the expression of CBFB following transfection with miR-145 mimic. (d) Protein expression of CBFB was evaluated by western blot analysis. (e) Expression of CBFB in hBMSCs was detected by RT-qPCR. *P < 0.05, **P < 0.01 and ***P < 0.001. CBFB, core-binding factor subunit β; miR-145, microRNA-145

To further explore the possible regulatory mechanisms of circ_0062582 and miR-145 in the differentiation of hBMSCs, the TargetScan online bioinformatics software was used to the prediction of the targets of miR-141-3p. It was found that CBFB could be a potential target gene of miR-145 (Figure 3a). The result of dual-luciferase assay experiments showed that miR-145 could directly target CBFB (Figure 3b). RT-qPCR and WB were employed to evaluate the expression of CBFB. It was observed that CBFB was downregulated following transfection with miR-145 overexpression plasmid (Figure 3c and 3d). In addition, the expression of CBFB was upregulated over time in hBMSCs treated with OM (Figure 3e). Generally speaking, CBFB was a direct target gene of miR-145 in hBMSCs.

**Effect of circ_0062582 on the osteogenic differentiation of hBMSCs by regulating miR-145/CBFB pathway**

**Figure 4.**
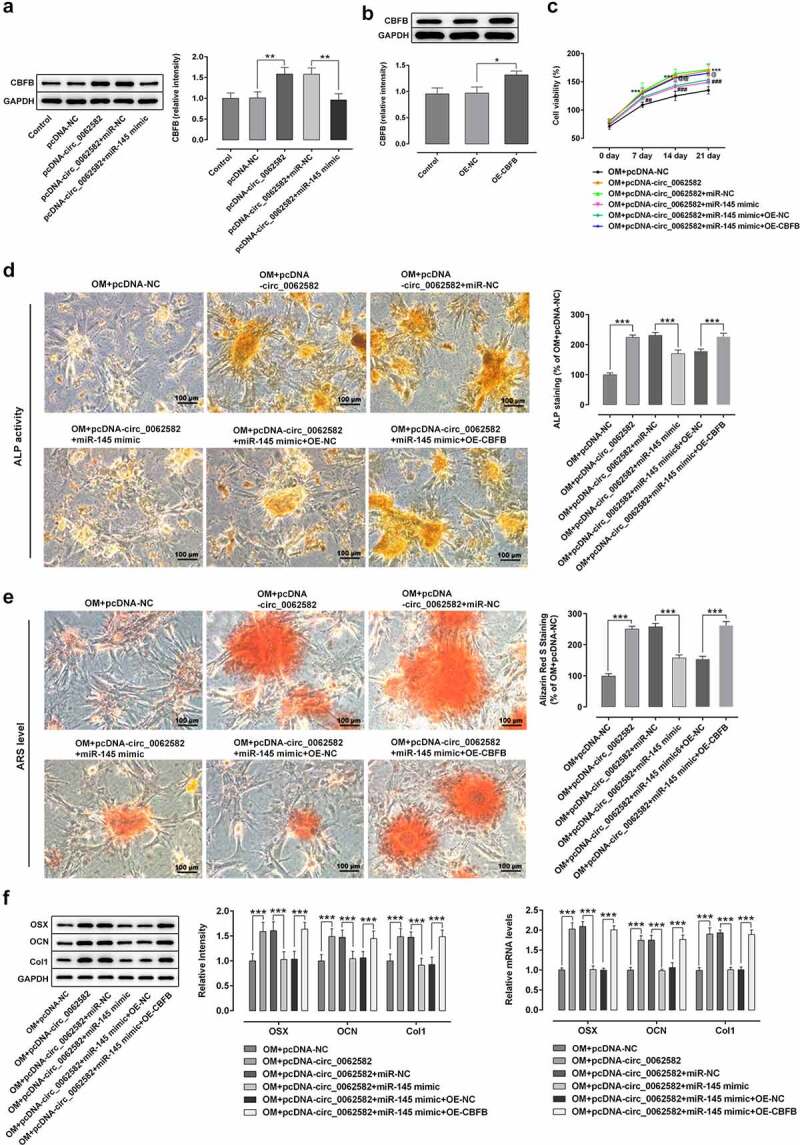
**Effect of circ_0062582 on the osteogenic differentiation of hBMSCs by regulating miR-145/CBFB pathway**. (a) Expression levels of CBFB in hBMSCs. (b) Expression levels of CBFB in hBMSCs following transfection with the CBFB overexpression vector. *P < 0.05, **P < 0.01. (c) Viability of hBMSCs. ***P < 0.001 vs. OM+pcDNA-NC; ^##^P < 0.01 and ^###^p < 0.001 vs. OM+pcDNA-circ_0062582+ miR-NC

^@@^P < 0.01 and ^@@@^P < 0.001 vs. OM+pcDNA-circ_0062582+ miR-145 mimic+OE-NC. (D) Alkaline phosphatase staining was used to detect the differentiation activity of hBMSCs. Magnification, x200. (E) Alizarin red staining was used to detect the mineralization levels of hBMSCs. Magnification, x200. (F) Protein expression levels of OSX, OCN and COL1. ***P < 0.001. CBFB, core-binding factor subunit β; hBMSCs, human bone marrow mesenchymal stem cells; OSX, osterix; OCN, osteocalcin; COL1, collagen type 1.

At last, rescue experiments were conducted to clarify the molecular mechanisms. WB and RT-qPCR were adopted for detecting the expression of CBFB in each group. Results indicated that CBFB expression was upregulated in the NC group following overexpression of cir0062582, while it was downregulated following transfection with miR-145 overexpression plasmid (Figure 4a). Next, CBFB overexpression plasmids were successfully constructed and results exhibited that the level of CBFB was higher in OE-CBFB group as comparison to the OE-NC group (Figure 4b). The result of CCK-8 assay showed that the promoting effect of circ_0062582 on the cell viability could be reduced by miR-145 overexpression, while these changes could be reversed by overexpression of CBFB (Figure 4c). ALP assay and alizarin red staining revealed increased cell differentiation after CBFB overexpression (Figure 4d and 4f). WB and RT-qPCR were used to detect the protein expression levels of OSX, OCN and COL1, which were observed to be notably upregulated in cells overexpressed by CBFB (Figure 4g). Taken together, circ_0062582 could regulate the osteogenic differentiation of hBMSCs by modulating miR-145/CBFB pathway.

## Discussion

In the present study, circ_0062582 was upregulated in the hBMSCs treated with OM. Overexpression of circ_0062582 significantly promoted cell proliferation, osteogenic differentiation and the expressions of osteogenic marker proteins. In addition, circ_0062582 directly targeted miR-145. MiR-145 upregulation conspicuously suppressed the effect of overexpression of circ_0062582 on the proliferation and osteogenic differentiation in hBMSCs. Lastly, CBFB served as the target gene of miR-145. These above activity changes could be partially reversed by overexpression of CBFB.

Osteoporosis, a degenerative disease caused by a variety of risk factors, is characterized by the loss of bone mass and the degeneration of bone microstructure, which will eventually lead to bone brittleness [23]. As an important component of bone marrow matrix, BMSCs are an important source of cells for osteogenic differentiation and bone formation in human body, which also have the potential to differentiate into osteoblasts in vitro [24]. The weakened osteogenic differentiation of BMSCs is accompanied by the loss of bone mass, which will further result in osteoporosis [25]. However, there are few studies focusing on the treatment of osteoporosis with BMSCs. CircRNA is a non-coding RNA that is present in large quantities in mammalian cells [26]. Furthermore, circRNAs have miRNA response elements that can bind to or release large amounts of miRNA in real time and, thus, effectively regulate gene expression [27,28]. circRNA can be used as a biomarker for osteoporosis, and a potential circRNA has been identified as a biomarker in the peripheral blood mononuclear cells of patients with postmenopausal osteoporosis [15]. One previous study has demonstrated that circ_0062582 expression is markedly downregulated in osteoporosis samples [16]. However, no research has shown the role and potential mechanism of circ_0062582 in osteoporosis. In this study, circ_0062582 overexpression was demonstrated to promote the differentiation and proliferation of hBMSCs.

MiRNAs are a class of non-coding RNAs 19–25 nucleotides in length that control gene expression at the post-transcriptional level [29]. miR-145 is a miRNA that has been widely reported to be abnormally expressed in a variety of human cancer types, including lung, prostate and breast cancer [20,30]. Additionally, emerging evidence supports that miR-145 expression was downregulated in osteosarcoma cells and tissues, and served a crucial role in osteocyte function [31,32]. In this study, overexpression of miR-145 suppressed the differentiation of the hBMSCs. Furthermore, the binding sites of miR-145 and CBFB were predicted using StarBase database. A number of studies have suggested that CBFB is required for osteoblast differentiation, as well as chondrocyte proliferation and maturation [33,34]. CBFB level was elevated over time with increasing cell differentiation. Following overexpression of CBFB, cell differentiation was enhanced in the OM + pcDNA-circ_0062582 + miR-145 group compared with the control group.

OSX is a transcription factor involved in the regulation of osteoblastic differentiation of cells that can specifically regulate the transcription of various osteogenesis-related genes. OSX expression is a requisite for osteoblasts to differentiate into osteoblasts as deletion of OSX gene will end the differentiation of osteoblasts [35,36]. COL1 is a specific collagen secreted by osteoblasts and the main component of bone organic matter [37]. The content of COL1 not only reflects the mature state of osteoblasts, but also affects the strength of bone, which serves an important role in all stages of osteoblast differentiation [38]. Known as bone Y-carboxyglutamate protein, OCN is an important bone matrix protein synthesized and secreted by osteoblasts [39]. It is a specific non-collagen matrix protein and a characteristic marker protein of osteocytes. Its distinctive producement means by mature differentiated osteocytes renders it effective in serving as a marker of osteocyte differentiation and maturation and regulating the process of bone mineralization [40]. The present study demonstrated that, following the overexpression of circ_0062586, the expression levels of OSX, OCN and COL1 were enhanced. After overexpression of miR-145, the protein expression levels were significantly decreased as comparison to the OM + pcDNA-circ_0062582 + miR-NC group. Finally, following overexpression of CBFB, the protein expression levels were upregulated compared with the OM + pcDNA-circ_0062582 + miR-145 mimic + overexpression-NC group. These results were consistent with those of ALP and alizarin red staining.

## Conclusion

In summary, this study shows that circ_0062582 affects osteoporosis by regulating CBFB/miR-145, providing the first line of evidence to clarify the underlying regulatory mechanisms of circ_0062582 in the development of osteoporosis *in vitro*. Our findings provides a theoretical foundation for circ_0062582 as a novel diagnostic biomarker and therapeutic target to improve osteogenic differentiation in osteoporosis. However, this study is an *in vitro* study and no *in vivo* experiments has been performed, which constitutes a limitation of the present study and the requirement of further in-depth investigations in the next studies.

## Supplementary Material

Supplemental Material

## Data Availability

All data generated or analyzed during this study are included in this published article.

## Highlights

1. Circ_0062582 expression was upregulated during osteogenic differentiation of hBMSCs.

2. Circ_0062582 acts as a sponge of miR-145 in hBMSCs.

3. CBFB is a target of miR-145 in hBMSCs, and positively regulated by circ_0062582.

4. Circ_0062582 promotes differentiation of hBMSCs by regulating miR-145/CBFB axis.
