# Supplementary figures and images for: Circular RNA_0062582 promotes osteogenic differentiation of human bone marrow mesenchymal stem cells via regulation of microRNA-145/CBFB axis
Source: Bioengineered. 2021 May 23;12(1):1952–63. doi: 10.1080/21655979.2021.1921553 (PMC8806255; doi:10.1080/21655979.2021.1921553)

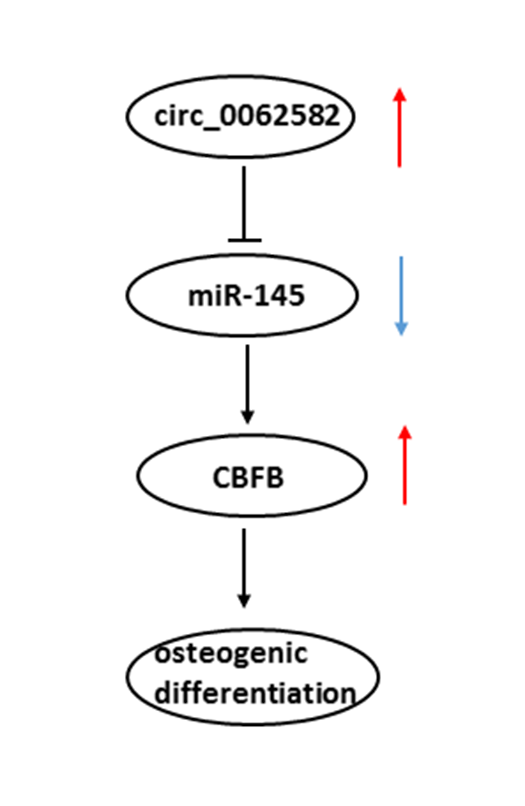

Supplement: Supplemental Material [file KBIE_A_1921553_SM0385.tif]
